# *Frankenstein; or, the modern Prometheus*: a classic novel to stimulate the analysis of complex contemporary issues in biomedical sciences

**DOI:** 10.1186/s12910-021-00586-7

**Published:** 2021-02-23

**Authors:** Irene Cambra-Badii, Elena Guardiola, Josep-E. Baños

**Affiliations:** 1 Chair in Bioethics, Centre d’Estudis Sanitaris I Socials (CESS), Universitat de Vic – Universitat Central de Catalunya, Carrer Miquel Martí i Pol, 1, 08500 Vic, Spain; 2 School of Medicine, Universitat de Vic – Universitat Central de Catalunya, Vic, Spain

**Keywords:** Health sciences, Science fiction, Bioethics, Frankenstein, Scientists’ responsibility, Teaching

## Abstract

**Background:**

Advances in biomedicine can substantially change human life. However, progress is not always followed by ethical reflection on its consequences or scientists’ responsibility for their creations. The humanities can help health sciences students learn to critically analyse these issues; in particular, literature can aid discussions about ethical principles in biomedical research. Mary Shelley’s *Frankenstein; or, the modern Prometheus* (1818) is an example of a classic novel presenting complex scenarios that could be used to stimulate discussion.

**Main text:**

Within the framework of the 200th anniversary of the novel, we searched PubMed to identify works that explore and discuss its value in teaching health sciences. Our search yielded 56 articles, but only two of these reported empirical findings. Our analysis of these articles identified three main approaches to using *Frankenstein* in teaching health sciences: discussing the relationship between literature and science, analysing ethical issues in biomedical research, and examining the importance of empathy and compassion in healthcare and research. After a critical discussion of the articles, we propose using *Frankenstein* as a teaching tool to prompt students to critically analyse ethical aspects of scientific and technological progress, the need for compassion and empathy in medical research, and scientists’ responsibility for their discoveries.

**Conclusion:**

*Frankenstein* can help students reflect on the personal and social limits of science, the connection between curiosity and scientific progress, and scientists’ responsibilities. Its potential usefulness in teaching derives from the interconnectedness of science, ethics, and compassion. *Frankenstein* can be a useful tool for analysing bioethical issues related to scientific and technological advances, such as artificial intelligence, genetic engineering, and cloning. Empirical studies measuring learning outcomes are necessary to confirm the usefulness of this approach.

## Background

In the last two centuries, scientific discoveries and technological innovations in biomedical sciences have improved the lives of most human beings immensely. However, education in the health sciences often fails to analyse the myriad consequences of scientific and technological advances from a bioethical point of view. In part, this failure derives from the compartmentalization of higher education. Bioethics is classified as a branch of moral philosophy, which is considered to lie in the sphere of the humanities rather than in the sphere of science and technology, and health sciences education largely ignores the humanities [1–6]. Moreover, traditional teaching methods like lectures are poorly suited to teaching issues related to bioethics, such as compassionate care or appropriate relationships among health professionals, patients, and society, which require active pedagogical techniques that help students develop critical thinking skills and problem-solving competences [7, 8].

Literature can help students appreciate the complexity of biomedical scenarios and improve their understanding of illness [9]. Various authors have suggested that literature can enhance future health professionals’ reflective thinking and improve their ability to analyse biomedical issues scientifically and honestly [10–16].

Mary Shelley’s *Frankenstein; or, the modern Prometheus*, published in 1818, is one of the most influential works in the history of English literature. It has influenced scientific thinking [17, 18] and has become a modern myth [17, 19–24]. Its value lies not only in its literary qualities, plot, and characters, but also in its reflective focus and compassionate approach to the character of Frankenstein’s creature [25].

Mary Shelley’s novel has come to be considered a canonical work. Its literary value and importance for science perdure today, more than 200 years after its first publication. Its place in the canon is ensured by its inclusion in educational curricula, especially in higher education [26], just as its place in popular culture is ensured by cinematic adaptations—especially James Whale’s (1931) and Kenneth Branagh’s (1994) versions, known worldwide. The current paper aims to examine the value of this work for ethicists and health sciences students, beyond popular culture or critical acclaim.

In a previous paper [27], we presented a content analysis of articles in the scientific literature that used the novel to discuss issues related to ethics, bioethics, science, technology, or medicine. We concluded that these articles focused mainly on Dr Frankenstein’s personality and scientific research rather than on ethical aspects related to his research or to the results of this research. Most of the papers analysed dealt with the importance of *Frankenstein* for reflecting science [17, 28–31], scientists [20, 32], the limits of scientific activity [22, 24, 33], and the need for peer review in research [32, 34].

In the current article, we review the literature on *Frankenstein* in the medical humanities and health sciences and propose different ways that Shelley’s novel can be used in teaching future health professionals.

## Main text

Searching PubMed using the term *Frankenstein* combined with ethics, bioethics, science, technology, medicine, education, and/or medical humanities yielded only two articles that reported empirical results about using the novel to teach health sciences. In the first, Koren and Bar [28] used a closed questionnaire, an essay, and a semi-structured collective interview about literary works including *Frankenstein*, *The Physicists*, *Gulliver’s Travels*, *Jurassic Park*, *Faust*, *Microbe Hunters*, *Galaxies*, and *Wrinkles in Time* to assess Israeli high-school students’ attitudes towards science and scientists. They found that *Frankenstein* was associated with the stereotypical “mad scientist”. In the interviews, a few students justified Frankenstein’s behaviour, but their ambivalent opinions were evident in sentences like “his intentions were relatively good to help the doctors and humanity afterwards he tried to fix it. But it was kind of too late.” (p.156).

In the second, Reginato et al. [1] used the field diary of a first-year biomedical sciences classroom in Brazil to evaluate the impact that reading and discussing *Frankenstein* had on their students in the context of a course that aimed to promote internal reflection about knowledge and the concept of science, responsibility and bioethics, and dehumanization in research. Students’ reflections focused mainly on two aspects: issues to be considered in science education beyond technical aspects, particularly the moral and ethical responsibility in research, and the influence of scientists’ actions in society.

Given the scarcity of research exploring the value of using *Frankenstein* in health sciences education, we sought to identify themes in the novel that would be of interest in this field and to develop ways that the novel could be exploited for teaching. Our critical analysis of the academic papers suggested three broad approaches: literature and science, bioethical dilemmas in research, and the need for empathy and compassion in medical care and research.

### Literature and science through *Frankenstein*

Mary Shelley’s *Frankenstein* is usually classified in the science fiction genre because it provides a critical vision of the future resulting from technoscientific advances [35]. However, the term science fiction was not coined until a century after Shelley’s novel was published. Frankenstein might also fit in the fiction-about-science genre [20] or even the science-in-fiction genre [36], where scientific facts are plausibly incorporated into fictional narratives to probe ethical dilemmas that might otherwise be difficult to tackle. Regardless of how we classify it, two hundred years after its initial publication, *Frankenstein* continues to lend itself to a critical analysis of science, knowledge, and responsibility. We will explore the use of literature for teaching health science students through three approaches.

First, discussions about whether the novel fits better in the science fiction or science-in-fiction genre can tackle various questions: What is science fiction literature? What are the science fiction elements in *Frankenstein*? What image of the future does the novel put forth? What other science fiction novels could be read together in the same context? (Answers to this question might include H. G. Wells’ *The Island of Dr Moreau* for considering human research or Aldous Huxley’s *Brave New World* for analysing human values). What films could be used to explore science fiction? (Answers to this question might include *Matrix* to review the fear of the technology beyond human control or *The Fly* to analyse research on human subjects).

Second, it could be interesting to analyse the historical and scientific context in which *Frankenstein* was written and its connections to literary works and later scientific developments. Along these lines, learning about the author’s private life and the scientific sources that inspired the novel could also be interesting. In the summer of 1816, Mary Shelley stayed in Villa Diodati, close to Lake Geneva, with her lover and future husband, Percy Shelley, her step-sister, Jane Clairmont, and her lover, Lord Byron and his personal physician John William Polidori. Bad weather confined them to the villa, where they had long conversations that served as inspiration for the scenes of galvanism and resuscitation in the novel [37]. It is fascinating to explore the state of scientific knowledge in the early nineteenth century to see how recent discoveries and currents of thought were adapted and incorporated into the novel, including Volta’s experiments with electricity; Galvani, Aldini, Bichat, and Weinhold’s investigations into electricity in animals and human beings and the influence of vitalism; Erasmus Darwin and the voluntary motion of vermicelli in a glass case; the antecedents of the alchemy of Paracelsus, Agrippa, and Albertus Magnus; Halley's magnetic theories; and the chemical processes of Davy and Bichat, some of which are explicitly mentioned in Mary Shelley’s introduction to the novel, written in 1831 [22, 23, 31, 38–49]. This approach shows the deep connections between the history and philosophy of science and bioethics and the health sciences. Learning about the scientific and sociohistorical context at the time the novel was written can help students understand the novel more deeply and gain insight into the relationships between the humanities and science. In this sense, it can be interesting to work not only with the content of the literary work, but also with the context in which it was created.

Third, *Frankenstein* can be used to discuss some issues in gender ethics in literature and science, beginning with its authorship and the circumstances of its publication. Mary Shelley’s name did not appear until the second edition of the novel, published in 1831. The first edition was published anonymously, and readers ascribed it to her husband, the romantic poet Percy B. Shelley, who had written the preface. Given the position of women in society at the beginning of the nineteenth century, in her preface of 1831, Mary Shelley took pains to explain how she was capable of producing a complete story without any help.

Gender roles and representations in the novel are also well worth analyzing. As previous researchers have pointed out, the novel seems to delimit two gender spheres: a masculine sphere that is scientific, ambitious, rational, active, and public, contrasting with a feminine one that is emotional, passive, and domestic [50–53]. Especially for teaching health science students, it can be interesting to discuss how the novel is narrated through the two male protagonists’ letters or diaries, while the female characters’ point of view is only intimated through two letters Elizabeth wrote to Victor before he created the creature. Not only is the human female perspective absent, however: the female creature, unlike her male counterpart, is not given the opportunity to discuss her plight in a monologue. To recover the voice of women, health science students can role play different scenarios incorporating the female characters’ views. Bibliographic searches about female scientists in Mary Shelley’s time can further enrich the discussion.

Finally, to discuss the relationship between literature and science, the narrative can be analysed in conjunction with other works and myths to explore the synergy between literature and science. The *Frankenstein* story has transcended the novel to become a modern myth [2, 17, 19–24, 49, 54, 55], building on the representational force of Prometheus and the Christian myths found in the novel. The reference to the myth of Prometheus explicitly present in the title is obvious, but the *Frankenstein* story also has connections with the Golem myth, inviting reflection on differences between the animate and the inanimate and between the human and inhuman. *Frankenstein* can also be considered together with Goethe's *Faust*, a scientist with uncontrollable ambitions, and with Stevenson’s *Dr Jekyll and Mr. Hyde*, another paradigm of human duality. The characters and plots allow us to reflect on the limits of human behaviour, what is and what is not permissible in scientific research, and whether these bioethical limits are time-dependent or should never be violated.

Just as *Frankenstein* was inspired by earlier myths, the story can also inspire new myths. The novel could be considered a template for science narratives, a social construction that helps people make sense of science and conceptualize its social and technical implications [17]. Nagy et al. [19] refer to the Frankenstein myth’s association with bad or dangerous science as a stigma that continues to haunt scientists. Thus, it would be useful to study whether students could identify negative images of science and scientists in *Frankenstein*, in other works of fiction, and in history as well.

### Bioethics dilemmas in research

*Frankenstein* is a good tool to examine the stereotype of the mad scientist [19–21, 54–56]. This stereotype calls attention to the risks associated with unsupervised research and can shed light on the evolution of society’s perception of science and scientists. Consciously or unconsciously, this stereotype is related to considerations of the bioethical limits of scientific research and the dilemmas that stem from scientific discoveries and technological advances. *Frankenstein* can help spark debate and focus discussion about these topics in health sciences classes.

Interestingly, as Nagy et al. [19] point out, the word scientist is not mentioned in the novel, because when *Frankenstein* was written, there was no term to refer to people who were dedicated to science. Victor Frankenstein nevertheless acts like a scientist, experimenting to gain new knowledge. Thus, despite the lack of references to the scientific profession, *Frankenstein* illustrates the power that comes with knowledge (scientific in this case), and this is one of the “lessons” that other authors have ascribed to the novel [20]. As Victor Frankenstein says, “Learn from me, if not by my precepts, at least by my example, how dangerous is the acquirement of knowledge, and how much happier that man is who believes his native town to be the world, than he who aspires to become greater than his nature will allow” [57].

Shelley’s work can also be analysed in relation to concerns about scientific and technological advances, their impact on human life, and the bioethical limits of current research. Indeed, the word *Frankensteinian*, when applied to practices in health sciences, refers to the risk involved in transgressive actions carried out without adequate prior consideration. In a sense, this meaning was articulated with Gaylin's [18] *Frankenstein factor*, a construct referring to the fear of poorly understood technological sophistication and the apprehension that technology can change the “nature” of species. Indeed, the name Frankenstein has often been used pejoratively as a warning in such scientific controversies [17, 33]. On the other hand, *Frankensteinian* is also used to refer to an attitude that values pure science or technology over reflection [58], a practice that is widespread not only in science, but also in education.

Names like “Frankenscience” [59], “Frankenfood” [60], or “Frankenstein syndrome” [61, 62] create cultural frames for viewing scientific enterprises and procedures in very specific and visceral ways that imply premeditated actions to alter nature [31, 63, 64]. These terms have being applied as warnings against “playing God” in applying practices such as transplantations [49]; robots, androids, and artificial intelligence in general [65, 66]; genetic engineering, gene therapy, or genomic editing with CRISPR-9 [24, 31, 67]; cloning [45]; and non-human bioengineered species [68].

Table 1 lists some papers that we consider especially useful for stimulating discussion about Frankenstein stigmas and current issues in science. Reflecting on these sources and some concrete case studies can help students appreciate the challenge of determining the limits and responsibilities of contemporary scientists in a variety of scenarios related to the fields mentioned above, as well as to others such as assisted reproductive technology, medical prolongation of life, use of organs and human tissues, the horizon of concern for eugenics [69], and the consequences that oppression and segregation have on human beings [20, 70].

**Table 1:** Some suggested readings to stimulate discussion on Frankenstein stigmas and ethical issues in current science

• *Robots, androids, and artificial intelligence in general:* Fell J. Could current experiments in science and technology lead to the creation of a modern-day Frankenstein's monster? Engin Technol. 2016;11(6):24–28
• *Genetic engineering or gene therapy, genomic editing with CRISPR-9:* Brokowski C, Adli M. CRISPR ethics: moral considerations for applications of a powerful tool. J Mol Biol. 2019 Jan 4;431(1):88–101
• *Cloning:* Jensen E. The Dao of human cloning: utopian/dystopian hype in the British press and popular films. Public Underst Sci. 2008 Apr;17(2):123–143
• *Nonhuman bioengineered species*: Hyun I. What’s wrong with human/nonhuman chimera research? PLoS Biol. 2016;14(8):e1002535
• *Assisted reproductive technology:* Ten Have H. (1995). Letters to Dr Frankenstein? Ethics and the new reproductive technologies. Soc Sci Med. 1995;40(2):141–146

Health science students can compare the contents of these papers to Frankenstein’s story or choose a recent controversial scientific advance and analyse the bioethical limits that could be involved and possible connections with *Frankenstein*. Furthermore, they could consider whether these warnings are based on moral opinion or on scientific assertions, taking into account the specific social context [71] and predictions of how these issues might be viewed in the future, while bearing in mind that moral values might be temporal [72].

The debate about creating life and the morality of “playing God” could lead into lessons about scientific ambition and ethical responsibilities in scientific advances [22]. Beyond technical and moral questions, “it is the inherent nature of science to push boundaries, discover new things, and commit overreach” [19], and students need to learn the importance of constant review of research and of supervision and feedback from expert colleagues and the general public. It is important to remember that Frankenstein's work is hidden, not shared with others, and removed from society [29, 48].

It can be useful to compare Frankenstein's acts and attitudes with those prescribed by current bioethical standards, principles, and guidelines for safe and ethical medical research. Although we must be cautious about judging the past based on current moral standards, this comparison can help students think about bioethics. They can identify the aspects of scientific research that were insufficiently protected in Frankenstein’s experiment and discuss the ethical dilemmas that biomedical advances imply for the present and future of humanity. They can also discuss to what extent views of events depend on the viewers’ perspective and the importance of historical context for bioethical principles. Table 2 summarizes a proposal for an activity relating Frankenstein to the bioethics of some fields of research.

**Table 2:** An example of how *Frankenstein* can be used to discuss the bioethical limits of some contemporary scientific discoveries

Teachers	Students
*Before the session*	
Definition of the main objective of the activity and selection of students’ readings	Reading of *Frankenstein*, full novel or selected chapters. If selected chapters, teachers can assign different chapters to different groups of students to enrich the proposals and improve the discussion
Preparation of a reading guide for students	Asking students to read paper(s) focused on the objective of the activity
	Viewing Kenneth Branagh’s 1994 film *Mary Shelley’s Frankenstein*
	Reviewing background information on the bioethical limits of scientific research
*During the session*	
Introduction of the historical and literary context of *Frankenstein*	Identifying the specified points related to the activity in *Frankenstein* and in the assigned papers
Explanation of the main objective of the activity	General discussion
Posing questions, such as: • How did advances in biomedical research happen? • Where did these advances occur? • What impact did they have on human health? • How were scientific advances received at the time they were achieved? • How should we deal with unintended consequences? • Where are the limits in human research? • What makes a scientist responsible? • What should Frankenstein have done?	Drawing conclusions
Moderation of students’ participation	
*After the session*	
Writing a brief report about the execution of the activity and to what extent the teaching objectives were achieved	Writing a short essay about how the relations between *Frankenstein* and current biomedical advances

### The need for empathy and compassion in medical care and research

Beyond the distinction between human and non-human creatures, this approach could delve into the themes related to the creation of living beings and scientists’ responsibility for their creations, as well as these creatures’ place in society and how they should be cared for.

In the novel, only a blind man treats Frankenstein’s creature kindly, and the creature cannot understand why his creator and society reject him. The creature shows that he has emotions and explains his desire to be accepted. He articulates his need for the companionship of a creature like him, promising to disappear peacefully if Dr Frankenstein creates a female creature to live with him and vowing to wreak havoc if he does not. Dr Frankenstein has misgivings about this project and undertakes it reluctantly, out of guilt, only to renege on his promise and destroy this second creation before it is finished.

In addition to analysing scientists’ responsibilities toward their creations, this approach can be used to explore the doctor-patient relationship. Given that empathy is a complex phenomenon that involves the ability to experience and share others’ feelings [73, 74] and compassionate care requires empathy for those who are suffering and efforts to alleviate their suffering [75], the novel can be used to discuss both of these concepts.

*Frankenstein* is an epistolary novel, so it allows us access into different memories as narrators change throughout the text although, as mentioned above, female voices are notably absent. Subjective, reflective, and perspectival memories are presented, but we must bear in mind which character is speaking in the novel [20]. The creature’s perspective can be an interesting point to discuss, especially his monologue about love and company.

Victor Frankenstein has difficulties manifesting empathy for the creature he has created and providing it with compassionate care; in fact, he does everything in his power to avoid these responsibilities, despite the creature’s obvious need for humane treatment. Some scenes in Kenneth Branagh’s (1994) film *Frankenstein of Mary Shelley* (e.g., the creature’s monologue and Victor Frankenstein’s response) illustrate this point masterfully. It is important for students to make the connection between the emotions evoked in the story and bioethical responsibility and to realise that bioethical responsibility is inseparable from empathy. Exchanging opinions about Frankenstein’s responsibilities as a scientist regarding the protection of the creature and society can help them make this connection, and examining the creature’s plight can help students understand three empathic abilities that they will have to develop in their careers as health professionals [76]: the ability to understand the patients’ situations (including their perspectives and feelings), the ability to communicate that they understand this situation, and the ability to use their understanding of the patient’s situation to improve on it.

Another approach is to have students prepare a debate in which they take the roles of the two characters, focusing on their emotions, needs, and responsibilities. The class can also reflect on situations where they felt cut off from their peers by any kind of discrimination or segregation, the prejudices in our society, and how they could change them.

Our approach underlines the importance of responsibility, bioethics, and compassionate care in health sciences. These themes can also lead to a discussion of attitudes toward science and bioethics from the point of view of aesthetics—the emotions they evoke, their utility or harmfulness, and their effects on society, the environment, and the family. These discussions are especially important for showing the connections between science and empathy through examining the different roles and situations in the story, for example, by discussing the relationship between scientific advances and Victor Frankenstein’s and the creature’s feelings and emotions. Table 3 summarizes some teaching objectives that can be considered when using *Frankenstein* as pedagogical tool.

**Table 3:** Some examples of general teaching objectives when using *Frankenstein* as a pedagogical tool

• To acquire an overview of biomedical research and its relationship with human health
• To understand how biomedicine evolves over time
• To analyse the synergy between different aspects of society and advances in knowledge and between literature and science, as well as the interdependence of these elements
• To analyse the limits of biomedical research according to social norms
• To promote critical thinking and bioethical reflection on the limits of biomedical research
• To discuss ethical dilemmas that biomedical advances represent for the present and future of humanity
• To articulate how bioethical limits are related to empathy and compassionate care

## Conclusions

Our earlier findings indicated that the scientific literature on *Frankenstein* focused mainly on science and the personality of the scientist rather than on the creature he created or on ethical aspects of his research [27]. The current paper explores how *Frankenstein* can be used to help health sciences students learn about bioethical issues through exploring the connections between science and literature, the need for bioethical limits, and how those limits relate to empathy and compassionate care.

Professionals and students of the health sciences need to assess advances in biomedical research from a critical bioethical viewpoint. The rapid advance of new technologies and their impact on human beings necessitate a sound bioethical analysis and deep reflection on scientists’ and healthcare professionals’ responsibilities in their implementation. The humanities, and especially literature, offer a powerful tool for reflection, since the characters and stories allow us to discuss current problems that have already appeared in completely different contexts and thus avoid focusing the debate exclusively on a specific contemporary situation.

Mary Shelley’s novel can be a good tool for analysing issues such as scientific research, the nature of science, and bioethical conflicts. Two centuries after its first publication, this literary work is far from being outdated or obsolete. It remains a valuable tool for reflecting on personal and social limits, the connection between curiosity and scientific progress, and the scientist’s responsibility in research.

We propose an active and participatory approach to learning based on exploration through questioning rather than on supplying ready-made answers. *Frankenstein* can reaffirm its value as a case study in teaching and expand the role of literary sources in the education of health sciences students. Future research should include studies to collect empirical evidence about the actual pedagogical effectiveness of this approach.

## Data Availability

Not applicable.
